# Real-world sinonasal outcomes in patients with primary diffuse chronic rhinosinusitis treated with mepolizumab across polyp phenotypes

**DOI:** 10.3389/fimmu.2026.1776180

**Published:** 2026-02-04

**Authors:** Nitish Kumar, Evani Patel, Michael J. Marino, Devyani Lal

**Affiliations:** 1 Department of Otorhinolaryngology – Head and Neck Surgery, Mayo Clinic, Phoenix, AZ, United States; 2 Mayo Clinic Alix School of Medicine, Phoenix, AZ, United States

**Keywords:** biologics, efficacy, endoscopic sinus surgery, mepolizumab, sinusitis, switching

## Abstract

**Background:**

Mepolizumab blocks IL-5, targeting eosinophilic type-2 inflammation. Due to existing phenotype driven patient selection, evidence of its effectiveness in primary diffuse CRS and CRS without nasal polyps (CRSsNP) and in real-world populations is limited. Our objective was to evaluate the real-world effectiveness of mepolizumab in patients with primary diffuse CRS, regardless of nasal polyp status, and to assess outcomes across CRS phenotypes and prior surgical history.

**Methods:**

Adults with primary diffuse CRS treated with mepolizumab for ≥6 months were identified. Pre- and post-therapy outcomes included serum eosinophil counts, Lund-Mackay CT scores, Lund-Kennedy endoscopic scores, and SNOT-22 symptom scores. Subgroup analyses were performed by CRS phenotype (CRSwNP *vs*. CRSsNP) and prior endoscopic sinus surgery (ESS). Biologic switching and discontinuation were recorded.

**Results:**

Among 277 patients (mean age 60.8 ± 14.7 years; 54.9% female), 93.5% had type-2 comorbidities, 29.6% had CRSsNP, and 27.8% were ESS-naïve. The median duration of mepolizumab therapy was 31 months. Mepolizumab therapy significantly reduced serum eosinophils (median 0.57 to 0.07 ×10^9^/L, p<0.001) and Lund-Mackay scores (median 14 to 10, p<0.001), which was consistent across all subgroups. Improvements in SNOT-22 and endoscopic scores were modest and not consistently significant. CRSwNP patients were more likely to undergo ESS during therapy, but time to first post-therapy ESS was longer than in CRSsNP. There were no differences in likelihood of undergoing ESS during therapy, time to subsequent ESS, or oral corticosteroid use between prior ESS and ESS-naïve patients. Biologic switching occurred in 24.9%, and discontinuation in 16.6%, primarily due to disease recalcitrance.

**Conclusion:**

Mepolizumab effectively reduced systemic eosinophilia and radiographic disease burden in primary diffuse CRS, independent of phenotype or prior ESS. Symptom improvement was variable, highlighting the heterogeneity of clinical response. These findings support an endotype-driven approach to biologic therapy and suggest that IL-5 blockade may benefit selected CRSsNP patients.

## 1 Introduction

The introduction of biologic therapy has transformed the management of chronic rhinosinusitis (CRS) through targeted inhibition of key cytokines involved in type-2 inflammation. Mepolizumab, a humanized IgG1κ monoclonal antibody, selectively targets interleukin-5 (IL-5), thereby preventing IL-5–mediated eosinophil maturation, activation, and survival (1). In 2021, mepolizumab was approved by the U.S. Food and Drug Administration as an add-on maintenance therapy for adults with CRS with nasal polyps (CRSwNP) who have an inadequate response to standard medical therapy, with demonstrated efficacy in reducing nasal polyp burden and improving disease control (2). Clinically, mepolizumab has been preferentially utilized in patients with CRSwNP who also have eosinophilic comorbidities such as asthma, hypereosinophilia, eosinophilic granulomatosis with polyangiitis, and eosinophilic esophagitis (3).

IL-5 plays a central role in eosinophilic inflammation, and elevated tissue and circulating eosinophil levels have been associated with increased disease severity, postoperative recurrence, and corticosteroid dependence in CRS (4–6). Although nasal polyposis has traditionally served as a clinical surrogate for type-2 inflammation, emerging evidence suggests that immunologic endotypes may better predict response to targeted biologic therapies than phenotypes alone (7). Notably, mixed inflammatory patterns have been identified across CRS phenotypes, with up to 55% of patients with CRS without nasal polyps (CRSsNP) demonstrating a type-2–predominant inflammatory profile (7). These findings raise the possibility that biologic therapies directed against type-2 inflammation may benefit a subset of CRSsNP patients who would otherwise be excluded based solely on phenotype. Such patients with type-2 CRSsNP disease might have variable upstream cytokine expression, but targeting of downstream expression on IL-5 mediated eosinophilic inflammation could represent a more stable and measurable therapeutic target in these patients.

To date, most studies evaluating the efficacy of mepolizumab—including randomized controlled trials, retrospective cohorts, and real-world analyses—have focused almost exclusively on patients with CRSwNP, limiting insight into the potential role of IL-5 blockade in CRSsNP patients with underlying type-2 inflammation (2, 8–15). Real-world evaluation of eosinophil-targeted therapy across CRS phenotypes may therefore provide important evidence regarding its broader applicability.

Accordingly, the primary aim of this study was to evaluate the real-world effectiveness of mepolizumab in patients with primary diffuse CRS, irrespective of nasal polyp status. Secondary aims included comparison of outcomes between CRSwNP and CRSsNP phenotypes, assessment of treatment response in patients with and without a prior history of endoscopic sinus surgery (ESS), and evaluation of rates and reasons for biologic switching and discontinuation.

## 2 Methodology

Following approval from the institutional review board (IRB ID 24-010845), electronic health records from all Mayo Clinic centers (Arizona, Florida, and Rochester) along with Mayo Clinic Health System were retrospectively reviewed to identify adult patients (≥18 years) with primary diffuse CRS who were treated with mepolizumab (100 mg subcutaneous injection every 4 weeks) for a minimum duration of 6 months. Diagnostic criteria used for primary diffuse CRS was the one defined by the European Position Paper on Sinusitis (2020) as bilateral, multi-sinus involvement with no underlying secondary causes such as vasculitis, odontogenic infection, immunodeficiency, ciliary dysfunction, and neoplastic etiology (16). As a retrospective real-world analysis, initiation of mepolizumab, specialty involvement, and follow-up assessments were determined by routine clinical care rather than a standardized protocol, and mepolizumab may have been prescribed for asthma or other type-2 inflammatory indications rather than CRS specifically.

Demographic and clinical data extracted from individual health records included age, biological sex, CRS phenotype (CRSwNP or CRSsNP), presence of type-2 inflammatory comorbidities, date of mepolizumab initiation, number of ESS procedures prior to biologic initiation, duration of therapy, and reasons for biologic switching or discontinuation when applicable. Treatment response was assessed using available pre-therapy (within 3 months prior to mepolizumab initiation) and post-therapy (≥6 months after initiation or most recent assessment/last available follow-up while on therapy) measures, including total and rhinologic subdomain scores of the 22-item Sinonasal Outcome Test (SNOT-22) (17, 18), Lund-Kennedy endoscopic scores (19), Lund-Mackay computed tomography scores (20), and serum eosinophil counts (×10^9^/L). A minimum follow-up of 6 months was chosen to allow sufficient time for biologic effect on sinonasal inflammation and symptom burden. The frequency of short oral corticosteroid (OCS) courses and the need for ESS after initiation of mepolizumab were recorded as additional indicators of disease control.

Subgroup analyses were performed based on CRS phenotype (CRSwNP *vs*. CRSsNP) and prior surgical history (ESS-naïve *vs*. prior ESS). Statistical analyses were conducted using R software. Data distribution was assessed using the Shapiro–Wilk test. Continuous variables were summarized as mean ± standard deviation or median with interquartile range (IQR), as appropriate. Due to substantial missing longitudinal data and variability in timing of outcome assessments, paired analyses were not feasible; therefore, pre- and post-therapy outcomes were analyzed as independent samples using the Mann–Whitney U test. For non-continuous and categorial variables, Chi-square and Fischer’s exact tests were used as appropriate. A two-sided *p*-value < 0.05 was considered statistically significant.

## 3 Results

A total of 277 patients with primary diffuse CRS were included in the analysis. The mean age of the cohort was 60.8 ± 14.7 years, and 152 patients (54.9%) were female. Type-2 inflammatory comorbidities were present in 259 patients (93.5%), with asthma being the most prevalent (247 patients, 89.2%), followed by allergic rhinitis (72 patients, 25.9%). Eighty-two patients (29.6%) had CRSsNP. A history of ESS prior to mepolizumab initiation was present in 200 patients (72.2%), while 77 patients (27.8%) were ESS-naïve.

The duration of mepolizumab therapy ranged from 6 to 120 months, with a median duration of 31 months (IQR 48). While receiving mepolizumab, 58 patients (20.9%) underwent subsequent ESS; among these, 48 patients (17.3%) required one procedure, eight patients (2.9%) required two procedures, and two patients (0.7%) underwent three procedures. The remaining 219 patients (79.1%) did not require ESS after mepolizumab therapy initiation. Given the high prevalence of asthma in the cohort, mepolizumab initiation could have been frequently driven by non-CRS indications, and sinonasal outcomes should be interpreted as downstream observations rather than direct measures of CRS-targeted treatment efficacy.

Daily low-dose OCS therapy was used by 28 patients (10.1%), whereas 178 patients (64.3%) required short OCS courses, with a median of one course (IQR 3) during the treatment period. It is crucial here to acknowledge the inability to definitively attribute OCS use to CRS versus comorbid asthma. Given the retrospective nature of the study and reliance on clinical records, the primary indication for OCS use could not be reliably determined. This limits interpretation of OCS exposure as a CRS-specific disease severity marker.

The pre-therapy *vs*. post-therapy values of Lund-Mackay scores (median 14 [IQR 8.5] *vs*. 10 [IQR 6], *p* < 0.001) and total SNOT-22 scores (median 35 [IQR 27] *vs*. 29 [IQR 32], *p* = 0.034) were significantly higher. In contrast, changes in the rhinologic subdomain of the SNOT-22 were not statistically significant (median 16 [IQR 9.5] *vs*. 14 [IQR 14], *p* = 0.100). Serum eosinophil levels showed a marked and significant reduction from a median of 0.57 ×10^9^/L (IQR 0.7) pre-therapy to 0.07 ×10^9^/L (IQR 0.06) post-therapy (*p* < 0.001). No statistically significant differences were observed in Lund-Kennedy endoscopic scores (6 [IQR 7.5] *vs*. median 4 [IQR 5], *p* = 0.149). It is important to mention that the statistical results need to be interpreted with caution due to missing data leading to unequal and small sample sizes for some of the comparisons, especially in the following subgroup analyses. Baseline characteristics and overall outcomes are summarized in **Table 1**.

**Table 1:** Baseline characteristics and summary of observations in the study cohort.

Variables	Values	*p*-value
Total patients (N)	277	
Sex distribution (M/F)	125/152 (45.1%/54.9%)	
Mean age (± SD)	60.8 ± 14.7 years	
Patients with type-2 comorbidities (%)	259 (93.5%)	
* Asthma*	*247 (89.2%)*	
* Allergic rhinitis*	*72 (25.9%)*	
* Atopic dermatitis*	*4 (1.4%)*	
* Others*	*3 (1.1%)*	
CRS phenotype: CRSwNP/CRSsNP	195/82 (70.4%/29.6%)	
Patients with history of ESS (%)	199 (72.1%)	
Median ESS procedures before initiating mepolizumab	1 (IQR: 2)	
Patients who underwent ESS after initiating mepolizumab (%)	58 (20.9%)	
* 1 ESS*	*48 (17.3%)*	
* 2 ESS*	*8 (2.9%)*	
* 3 ESS*	*2 (0.7%)*	
Median time to first ESS after initiating mepolizumab therapy	11 months (IQR: 25)	
Patients using OCS during mepolizumab therapy (%)	206 (74.4%)	
* Low-dose daily therapy*	*28 (10.1%)*	
* Short course therapy*	*178 (64.3%)*	
Median frequency of short course OCS use	1 (IQR: 3)	
Median pre-therapy CT Lund-Mackay scores (IQR)	14 (8.5); n = 48	< 0.001*
Median post-therapy CT Lund-Mackay scores (IQR)	10 (6); n = 111	
Median pre-therapy endoscopic Lund-Kennedy scores (IQR)	6 (7.5); *n* = 30	0.149*
Median post-therapy endoscopic Lund-Kennedy scores (IQR)	4 (5); *n* = 86	
Median pre-therapy total SNOT-22 scores (IQR)	35 (27); *n* = 52	0.034*
Median post-therapy total SNOT-22 scores (IQR)	29 (32); *n* = 122	
Median pre-therapy rhinologic SNOT-22 scores (IQR)	16 (9.5)	0.100*
Median post-therapy rhinologic SNOT-22 scores (IQR)	14 (14)	
Median pre-therapy serum eosinophil counts (IQR)	0.57×10^9^/L (0.7); *n* = 134	0.001*
Median post-therapy serum eosinophil counts (IQR)	0.07×10^9^/L (0.06); *n* = 193	

ESS: Endoscopic sinus surgery, OCS: Oral corticosteroids, IQR: Interquartile range, SNOT-22: 22-item sinonasal outcome test.

*Mann-Whitney U test.

### 3.1 Impact of mepolizumab therapy on primary diffuse CRSwNP and primary diffuse CRSsNP

Patients with CRSwNP were significantly more likely to have undergone ESS (83.1% *vs*. 46.6%, *p* < 0.001) and had more ESS procedures performed (median 1 *vs*. 0, p < 0.001) prior to mepolizumab initiation, and were also more likely to require ESS (24.6% *vs*. 12.2%, *p* = 0.031) with more ESS procedures performed during biologic therapy (median 0 *vs*. 0, p = 0.023) compared to those with CRSsNP. This difference, despite median procedures being 0 for both phenotypes, reflects the differences in the distribution (e.g., a higher proportion of CRSwNP patients undergoing one or more ESS procedures), rather than a difference in the median itself. Nine (4.6%) CRSwNP patients underwent > 1 ESS procedures after starting therapy compared to 2 (2.4%) CRSsNP patients. However, time to first ESS after initiating mepolizumab was significantly longer in the CRSwNP subgroup (median 12 *vs*. 4 months, *p* = 0.034). The proportion of patients requiring OCS during therapy did not differ between phenotypes (*p* = 0.990). Pre-therapy serum eosinophil levels were higher in the CRSwNP subgroup (median 0.67 *vs*. 0.42 ×10^9^/L, *p* = 0.045), whereas baseline SNOT-22, Lund-Mackay, and Lund-Kennedy scores were comparable between groups.

Within both phenotypes, significant post-therapy reductions were observed only for Lund-Mackay scores and serum eosinophil levels. No statistically significant pre- to post-therapy changes were detected in total or rhinologic SNOT-22 scores or Lund-Kennedy scores in either subgroup.

Also, there were no significant difference in biologic switching and discontinuation rates between these phenotypic groups.

Detailed subgroup analyses are presented in **Table 2**.

**Table 2:** Summary of observations in CRSwNP and CRSsNP subgroups receiving mepolizumab therapy.

Variable	CRSwNP	CRSsNP	*p*-value
Total patients (N)	195	82	
Sex distribution: M/F	85/110	39/43	0.635**
Mean age (± SD)	60.1 ± 14.8 years	62.3 ± 14.3 years	0.189*
Patients with type-2 comorbidities (%)	183 (93.8%)	77 (93.9%)	1.000**
* Asthma*	*173 (89.1%)*	*74 (91.4%)*	
* Allergic rhinitis*	*48 (24.6%)*	*24 (29.3%)*	
* Atopic dermatitis*	*4 (2.0%)*	*0*	
* Others*	*3 (1.5%)*	*0*	
Patients with history of ESS (%)	162 (83.1%)	39 (46.6%)	<0.001**
Median ESS procedures before initiating mepolizumab (IQR)	1 (2)	0 (1)	<0.001**
Median duration of mepolizumab therapy (IQR)	31 months (46.5)	30.5 months (47.5)	0.793*
Patients who underwent ESS after initiating mepolizumab (%)	48 (24.6%)	10 (12.2%)	0.031**
Median time to first ESS after initiating mepolizumab therapy (IQR)	12 months (25)	4 months (3.5)	0.034*
Patients using OCS during mepolizumab therapy	112 (57.4%)	52 (63.4%)	0.429**
* Low-dose daily therapy*	*16 (8.2%)*	*12 (14.6%)*	
* Short course therapy*	*96 (49.2%)*	*40 (48.8%)*	
Median number of short OCS courses (IQR)	1 (2.5)	1 (3)	0.990*
Patients switching mepolizumab (%)	41 (21.0%)	18 (21.9%)	0.991**
Patients discontinuing mepolizumab (%)	33 (16.9%)	13 (15.8%)	0.967**
Median pre-therapy CT Lund-Mackay scores (IQR)	14 (9); *n* = 35	11 (6.0); *n* = 13	0.268*
Median post-therapy CT Lund-Mackay scores (IQR)	10 (5); *n* = 85	8.5 (6.7); *n* = 26	
* p*-value of pre- *vs*. post-therapy comparison	0.003*	0.019*	
Median pre-therapy endoscopic Lund-Kennedy scores (IQR)	7 (8); *n* = 23	3 (6); *n* = 7	0.071*
Median post-therapy endoscopic Lund-Kennedy scores (IQR)	4 (6); *n* = 69	2 (4); *n* = 17	
* p*-value of pre- *vs*. post-therapy comparison	0.078*	0.999*	
Median pre-therapy total SNOT-22 scores (IQR)	35 (23); *n* = 35	38 (33); *n* = 17	0.482*
Median post-therapy total SNOT-22 scores (IQR)	29 (33.2); *n* = 100	34 (40); *n* = 22	
* p*-value of pre- *vs*. post-therapy comparison	0.062*	0.552*	
Median pre-therapy rhinologic SNOT-22 scores (IQR)	17.5 (9.5)	15.5 (9.25)	0.868*
Median post-therapy rhinologic SNOT-22 scores (IQR)	14.5 (13.7)	13 (17)	
* p*-value of pre- *vs*. post-therapy comparison	0.153*	0.398*	
Median pre-therapy serum eosinophil counts (IQR)	0.6 (0.7); *n* = 100	0.42 (0.55); *n* = 34	0.045*
Median post-therapy serum eosinophil counts (IQR)	0.06 (0.07); *n* = 134	0.07 (0.06); *n* = 59	
* p*-value of pre- *vs*. post-therapy comparison	< 0.001*	< 0.001*	

ESS: Endoscopic sinus surgery, OCS: Oral corticosteroids, IQR: Interquartile range, SNOT-22: 22-item sinonasal outcome test.

*Mann-Whitney U Test, **Chi-Square Test, ***Fischer’s Exact Test.

### 3.2 Impact of mepolizumab therapy on ESS-naïve and prior-ESS subgroups

Patients in the prior-ESS subgroup were more likely to have CRSwNP compared with ESS-naïve patients (81.0% *vs*. 44.2%, *p* < 0.001). No significant differences were observed between ESS-naïve and prior-ESS groups with respect to the proportion of patients undergoing post-therapy ESS, number of post-therapy ESS procedures, time to first ESS after biologic initiation, or OCS use. Pre-therapy SNOT-22, Lund-Mackay, and Lund-Kennedy scores were comparable between groups. There was no significant difference in the baseline serum eosinophil levels as well between both the groups.

There was a significant reduction in pre- *vs*. post-therapy serum eosinophil counts and Lund-Mackay scores in both prior-ESS and ESS-naïve subgroups. While median SNOT-22 scores numerically improved in both subgroups post mepolizumab therapy, these changes did not reach statistical significance, likely reflecting limited sample size, heterogeneous follow-up timing, and missing data. Similarly, Lund–Kennedy scores showed divergent non-significant trends between subgroups and should be interpreted cautiously given the very small number of patients with available endoscopic data, particularly in the ESS-naïve group. Full details are provided in **Table 3**.

**Table 3:** Summary of observations in ESS naïve subgroup and subgroup of patients with a history of prior ESS receiving mepolizumab therapy.

Variable	ESS naïve	Prior ESS	*p*-value
Total patients (N)	77	200	
Sex distribution: M/F	37/40	88/112	0.637**
Mean age (± SD)	60.2 ± 15.1 years	61.1 ± 14.6 years	0.778*
Patients with type-2 comorbidities (%)	73 (94.8%)	186 (93.0%)	0.787**
* Asthma*	*69 (89.6%)*	*177 (88.5%)*	
* Allergic rhinitis*	*25 (32.5%)*	*46 (23.0%)*	
* Atopic dermatitis*	*2 (2.6%)*	*2 (1.0%)*	
* Others*	*0*	*3 (1.5%)*	
CRS phenotype: CRSwNP/CRSsNP	34/43 (44.2%/55.8%)	162/38 (81.0%/19.0%)	<0.001**
Patients who underwent ESS after initiating mepolizumab (%)	21 (27.3%)	38 (19.0%)	0.080**
Median ESS procedures (IQR)	0 (1)	0 (0)	0.221*
Median time to first ESS after initiating mepolizumab therapy (IQR)	8 months (17)	12.5 months (25.5)	0.222*
Patients using OCS during mepolizumab therapy (%)	59 (76.6%)	112 (56.0%)	<0.001**
* Low-dose daily therapy*	*5 (6.5%)*	*23 (11.6%)*	0.310**
* Short course therapy*	*54 (70.1%)*	*89*	<0.001**
Median number of short OCS courses	1 (IQR: 3)	1 (IQR: 2)	0.181*
Patients switching mepolizumab (%)	16 (20.8%)	43 (21.5%)	1.000**
Patients discontinuing mepolizumab (%)	15 (19.5%)	36 (18.0%)	0.911**
Median pre-therapy CT Lund-Mackay scores (IQR)	14 (3); *n* = 11	12 (9); *n* = 37	0.146*
Median post-therapy CT Lund-Mackay scores (IQR)	10 (9); *n* = 29	10 (5); *n* = 82	
* p*-value of pre- *vs*. post-therapy comparison	0.001*	0.021*	
Median pre-therapy endoscopic Lund-Kennedy scores (IQR)	2 (4); *n* = 5	8 (7); *n* = 25	0.064*
Median post-therapy endoscopic Lund-Kennedy scores (IQR)	3 (4); *n* = 15	4 (6); *n* = 71	
* p*-value of pre- *vs*. post-therapy comparison	0.226*	0.075*	
Median pre-therapy total SNOT-22 scores (IQR)	35 (25.5.); *n* = 8	35 (28); *n* = 44	1.000*
Median post-therapy total SNOT-22 scores (IQR)	24 (23.5); *n* = 22	30 (34); *n* = 99	
* p*-value of pre- *vs*. post-therapy comparison	0.437*	0.080*	
Median pre-therapy rhinologic SNOT-22 scores (IQR)	16 (5.7)	16 (10.7)	0.822*
Median post-therapy rhinologic SNOT-22 scores (IQR)	12 (11.5)	14.5 (13.2)	
* p*-value of pre- *vs*. post-therapy comparison	0.621*	0.183*	
Median pre-therapy serum eosinophil counts (IQR)	0.64 (0.66); *n* = 36	0.52 (0.72); *n* = 98	0.806*
Median post-therapy serum eosinophil counts (IQR)	0.08 (0.06); *n* = 51	0.06 (0.07); *n* = 136	
* p*-value of pre- *vs*. post-therapy comparison	< 0.001*	< 0.001*	

ESS: Endoscopic sinus surgery, OCS: Oral corticosteroids, IQR: Interquartile range, SNOT-22: 22-item sinonasal outcome test.

*Mann-Whitney U Test, **Chi-Square Test, ***Fischer’s Exact Test.

### 3.3 Mepolizumab switching and discontinuation

Mepolizumab was switched to an alternative biologic agent in 69 patients (24.9%), most commonly due to disease recalcitrance (42 patients, 60.9%). Additional reasons included insurance denial and cost (two patients each) and arthralgia (one patient), while reasons were undocumented in 22 patients. Discontinuation of mepolizumab occurred in 46 patients (16.6%), primarily due to disease recalcitrance (17 patients, 40.0%). Other reasons included cost of therapy (6, 13.0%), denial by insurance (5, 10.9%), resolution of symptoms (5, 10.9%), loss to follow-up (4, 8.9%), adverse effects including hypertension, constipation, and arthralgias (4, 8.9%), and logistic issues (2, 4.3%). The reasons for discontinuation were unknown in 3 patients.

## 4 Discussion

In this large real-world cohort of patients with primary diffuse CRS, we demonstrate that mepolizumab therapy is associated with significant reductions in circulating eosinophil levels and radiographic disease burden, irrespective of nasal polyp status or prior surgical history. Importantly, these objective improvements were observed across both CRSwNP and CRSsNP phenotypes, supporting the concept that immunologic endotype rather than visible phenotype may better predict response to eosinophil-targeted biologic therapy (21, 22). At the same time, improvements in patient-reported sinonasal symptoms and endoscopic findings were more modest and did not consistently reach statistical significance, highlighting the complexity of translating immunologic modulation into symptomatic benefit in heterogeneous real-world CRS populations. It is noteworthy that the use of mepolizumab in CRSsNP patients might be driven by the co-existent asthma, as CRSsNP is not yet the approved indication for mepolizumab therapy. However, this provides us the opportunity to observe the effectiveness of mepolizumab in managing sinonasal disease in patients with CRSsNP.

### 4.1 IL-5 blockade and objective disease control across CRS phenotypes

IL-5 plays a central role in eosinophil differentiation, activation, and survival, and eosinophilic inflammation has been strongly linked to disease severity, recurrence after surgery, and corticosteroid dependence in CRS (16, 23). In our cohort, mepolizumab therapy resulted in a profound and consistent reduction in serum eosinophil counts. This effect was observed regardless of CRS phenotype or prior ESS status, reinforcing the biological specificity and robustness of IL-5 blockade in suppressing systemic eosinophilia.

Concurrently, we observed significant reductions in CT Lund–Mackay scores following mepolizumab therapy across the overall cohort as well as within CRSwNP, CRSsNP, ESS-naïve, and prior-ESS subgroups. Radiographic improvement likely reflects a reduction in mucosal inflammation and tissue edema driven by eosinophilic infiltration. The consistency of this finding across phenotypes is particularly noteworthy, as CRSsNP has historically been considered less amenable to biologic therapy due to its perceived non–type-2 inflammatory profile (16). The lack of significant improvement in endoscopic scores could reflect a floor effect inherent to CRSsNP. Patients without nasal polyps generally demonstrate lower baseline endoscopic inflammatory scores (median pre-therapy score of 3 in our cohort), thereby reducing the measurable range for post-treatment improvement. In this context, even biologically meaningful reductions in mucosal inflammation may not translate into statistically significant changes in endoscopic scoring systems. Our data support emerging evidence that a substantial proportion of CRSsNP patients harbor type-2–predominant endotypes and may derive objective benefit from eosinophil-directed treatment (7).

### 4.2 Symptom response and the phenotype–endotype disconnect

Despite clear improvements in eosinophil counts and radiographic disease burden, changes in total and rhinologic SNOT-22 scores did not consistently reach statistical significance in the overall cohort or in subgroup analyses. This finding contrasts with randomized controlled trials of biologics in CRSwNP, where symptom improvement is often more pronounced, and underscores several important considerations inherent to real-world data (21, 24).

First, patient-reported outcome measures such as SNOT-22 are influenced by multiple factors beyond sinonasal inflammation, including asthma control, prior surgical scarring, neuropathic symptoms, and comorbid conditions (21). In a cohort with a high prevalence of asthma and extensive prior ESS, symptom burden may persist despite biologic-mediated reductions in eosinophilic inflammation. Second, the use of unpaired analyses, necessitated by missing longitudinal assessments, selection bias, and variable follow-up intervals, likely reduced statistical power to detect symptom changes. Third, IL-5 blockade primarily targets eosinophilic pathways and may have less impact on neurogenic inflammation or structural contributors to symptoms (16).

Interestingly, although rhinologic SNOT-22 scores did not show statistically significant improvement, the directionality of change favored post-therapy improvement across analyses. This suggests that selected patients may experience clinically meaningful benefits even when group-level differences fail to reach statistical significance. These findings reinforce the need for refined biomarkers and endotype-driven selection to better predict symptomatic response to biologic therapy in CRS. A potential area of investigation can be the outcomes in high-eosinophilia *vs*. low-eosinophilia patients, to further observe the differences of mepolizumab efficacy based on serum eosinophil levels which can contribute in improving our understanding of endotype driven mepolizumab response.

### 4.3 Impact of prior ESS and disease refractoriness

A large proportion of patients in our cohort had undergone ESS prior to initiating mepolizumab, like that previously reported in real-world studies (13), reflecting the refractory nature of disease in real-world biologic candidates. Patients with CRSwNP were significantly more likely to have undergone ESS both before and after starting mepolizumab, consistent with the more aggressive and recurrent disease course traditionally associated with nasal polyposis. Notably, although CRSwNP patients underwent ESS more frequently during biologic therapy, the time to first post-mepolizumab ESS was significantly longer compared with CRSsNP patients. This finding suggests that mepolizumab may delay surgical recurrence in CRSwNP, even if it does not fully eliminate the need for subsequent intervention.

When stratified by surgical history, both ESS-naïve and prior ESS patients demonstrated similar patterns of eosinophil and radiographic improvement, indicating that prior surgical intervention does not preclude biologic responsiveness. This observation is clinically relevant, as biologics are often introduced late in the treatment algorithm, after multiple surgical procedures. Our data support the use of mepolizumab as a disease-modifying therapy even in surgically naïve patients, while also highlighting that biologic therapy does not uniformly obviate the need for surgery.

### 4.4 Switching and discontinuation: real-world treatment durability

In this real-world cohort, nearly one-quarter of patients switched from mepolizumab to an alternative biologic and approximately one-sixth discontinued therapy, with disease recalcitrance emerging as the dominant driver of both treatment modification and cessation. This pattern aligns with growing real-world evidence in CRS and severe asthma populations demonstrating substantial interindividual variability in response to IL-5–targeted therapies, despite reductions in eosinophilic inflammation. While randomized trials have established the efficacy of mepolizumab in CRS, effect sizes are generally modest compared with biologics targeting upstream type 2 pathways, and eosinophil depletion alone may be insufficient to address the multifactorial pathophysiology of CRS, which includes epithelial barrier dysfunction, neurogenic inflammation, innate immune activation, and irreversible tissue remodeling (5, 14, 25, 26). Notably, switching was more common than complete discontinuation, suggesting that persistent inflammatory disease activity—rather than treatment futility—often prompted redirection toward alternative biologic targets, consistent with evolving precision medicine approaches in airway disease (12, 27, 28).

Non-clinical factors, including insurance denial, cost, and logistical barriers, also contributed meaningfully to both switching and discontinuation, underscoring the impact of real-world access constraints that are underrepresented in clinical trials but increasingly recognized in observational biologic studies (29). Adverse effects leading to discontinuation were uncommon and consistent with the established safety profile of mepolizumab, indicating that tolerability was not a major limiting factor in long-term use (30). Finally, undocumented reasons for treatment modification in a minority of patients reflect inherent limitations of retrospective real-world data, particularly in multimorbid populations, but do not detract from the overall observation that incomplete disease control, rather than safety concerns, is the principal driver of mepolizumab switching and discontinuation in routine CRS practice.

It is noteworthy that the rates of adverse effects and discontinuation observed in our study were higher than previously reported rates, which could be a function of relatively much longer follow-up period and the real-world nature of our study with varying characteristics of the study cohort compared to clinical trials (2, 10, 12).

### 4.5 Strengths and limitations

The strengths of this study include its large sample size, multicenter real-world design, longer follow-up durations compared to existing studies (13), inclusion of both CRSwNP and CRSsNP phenotypes, and comprehensive assessment of objective outcomes, patient-reported measures, surgical utilization, and treatment durability. Few studies to date have examined IL-5 blockade across CRS phenotypes in a real-world setting with this level of granularity.

This study lacks standardized follow-up intervals and systematic outcome collection, reflecting routine clinical practice across multiple centers. Additionally, given the predominance of asthma and other type-2 inflammatory comorbidities, mepolizumab could have been often initiated for non-CRS indications, with shared otolaryngology involvement in treatment decisions and follow-up. Consequently, observed changes in sinonasal outcomes cannot be attributed solely to CRS-directed biologic therapy and should be interpreted as descriptive real-world associations. The retrospective design and reliance on electronic health records resulted in missing and unpaired longitudinal data, limiting the ability to perform paired analyses and potentially underestimating treatment effects. Variability in timing of outcome assessments and indications for imaging or endoscopy may have introduced selection bias. While the Mann–Whitney U test does not require uniform follow-up timing, we acknowledge that variable assessment intervals may introduce heterogeneity in treatment response. However, this approach reflects real-world, sustained outcomes rather than short-term effects at a fixed time point. Additionally, tissue-level inflammatory biomarkers were not available, precluding direct endotypic confirmation. Finally, the absence of a control group limits causal inference.

## 5 Conclusions

In this large real-world cohort of patients with primary diffuse CRS, mepolizumab therapy was associated with significant reductions in systemic eosinophilia and radiographic disease burden, independent of nasal polyp status or prior ESS. These findings support an endotype-driven approach to biologic therapy in CRS, in which eosinophilic inflammation may be more relevant than traditional phenotypic classification.

Symptom improvement was more variable and did not consistently reach statistical significance, reflecting the heterogeneity of treatment response in real-world practice. Nevertheless, the observed objective disease modulation and delayed need for surgery in select patients suggest a meaningful role for IL-5 blockade in appropriately selected individuals. Future prospective studies incorporating molecular biomarkers and standardized outcome measures are needed to better define predictors of response and optimize personalized biologic therapy in CRS.

## Data Availability

The raw data supporting the conclusions of this article will be made available by the authors, without undue reservation.
